# Transmission of *Bamboo mosaic virus* in Bamboos Mediated by Insects in the Order *Diptera*

**DOI:** 10.3389/fmicb.2017.00870

**Published:** 2017-05-16

**Authors:** Kuo-Chen Chang, Ling-Teng Chang, Ying-Wen Huang, Yi-Chin Lai, Chin-Wei Lee, Jia-Teh Liao, Na-Sheng Lin, Yau-Heiu Hsu, Chung-Chi Hu

**Affiliations:** ^1^Graduate Institute of Biotechnology, National Chung Hsing UniversityTaichung, Taiwan; ^2^Institute of Plant and Microbial Biology, Academia SinicaTaipei, Taiwan

**Keywords:** insect transmission, *Diptera*, *Gastrozona fasciventris*, *Atherigona orientalis*, *Potexvirus*, *Bamboo mosaic virus*, bamboo

## Abstract

*Bamboo mosaic virus* (BaMV), a member of the genus *Potexvirus*, is the major threat to bamboo cultivation. Similar to most potexviruses, the transmission of BaMV by insect vectors has not been documented previously. However, field observations of BaMV disease incidences suggested that insect vectors might be involved. In this study, we aimed to investigate the possibility of insect-mediated transmission of BaMV among bamboo clumps, in order to provide further insights into the infection cycles of BaMV for the development of effective disease management measures. From the major insects collected from infected bamboo plantations, BaMV genomic RNAs were detected inside the bodies of two dipteran insects, *Gastrozona fasciventris* and *Atherigona orientalis*, but not in thrips (*Scirtothrips dorsalis*). Artificial feeding assays using green fluorescent protein-tagged BaMV virions revealed that BaMV could enter the digestive systems and survive in the regurgitant and excretion of the dipterans. BaMV RNA could be retained in the dipterans for up to 4 weeks. Insect-mediated transmission assays indicated that both dipterans could transmit BaMV to bamboo seedlings through artificially created wounds with low infection efficiency (14 – 41%), suggesting that the dipterans may mediate the transmission in a mechanical-like manner. These results demonstrated that dipterans with sponge-like mouthparts may also serve as vectors for at least one potexvirus, BaMV, among bamboo plants. The finding suggested that dipteran insect control should be integrated into the disease management measures against BaMV infections.

## Introduction

Bamboos are economically important crops for their broad applications in both agriculture and industry. However, the cultivation of bamboo crops is under the threat of a major pathogen, *Bamboo mosaic virus* (BaMV), a member of the genus *Potexvirus* in the family *Flexiviridae* (Lin et al., 1992). BaMV has been reported to infect different bamboos in Brazil, Taiwan, California and Florida (USA), Australia, Hawaii, and Mainland China (Lin et al., 1977, 1979, 1993, 1995, 2014; Lin and Chen, 1991; Elliot and Zettler, 1996; Dodman and Thomas, 1999; Nelson and Borth, 2011). At least 10 commercially cultivated species of bamboos are susceptible to the infections of BaMV (Lin et al., 1993). Typical symptoms of infected bamboos include mosaic or chlorotic streaks in between the veins of the leaves, necrotic tissues (brown to black spots or streaks) in the shoots and culms, aborted shoots, reduced vigor, and even death of the bamboo clumps (Lin et al., 1993; Hsu and Lin, 2004; Nelson and Borth, 2011). The necrotic tissues in the shoots greatly reduce the yield, quality, and value of the bamboo products, and are usually referred to by farmers as bamboo shoot “nails” due to the resemblance in appearance and texture. With a disease prevalence of about 70–80% in bamboo plantations in Taiwan (Lin et al., 1993), BaMV is recognized as one of the major limiting factors for bamboo cultivation.

Being a potexvirus, BaMV has the typical flexible filamentous virion structure (Dimaio et al., 2015), harboring a positive-sense, single-stranded RNA genome of about 6.4 kb in length, with a 5′-cap and a 3′ poly(A) tail (Lin et al., 1992; Chen et al., 2005). BaMV is believed to spread through vegetative propagation of bamboos using seedlings produced from BaMV-infected bamboo mother stocks, or mechanical transmission by BaMV-contaminated tools used in harvesting or pruning (Elliot and Zettler, 1996; Hsu and Lin, 2004; Nelson and Borth, 2011). However, the possible involvement of other transmission routes, such as via insect vectors, has not been ruled out.

**FIGURE 1:**
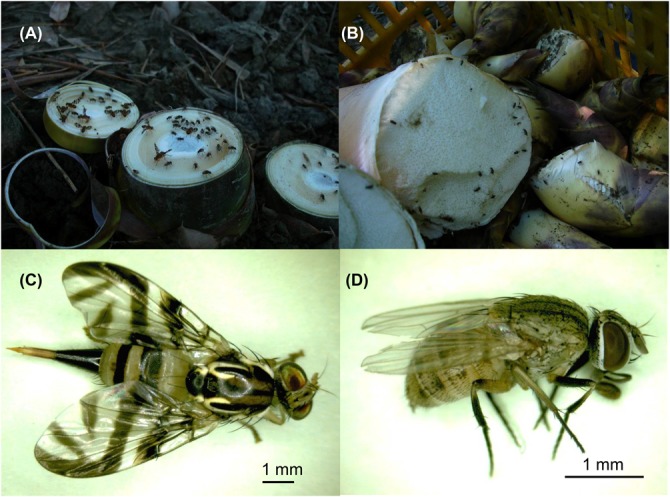
**The main insect species collected in bamboo plantations in southern Taiwan. (A)** After the harvesting of the bamboo shoots, the cut surfaces exposed on the ground attracted many dipteran insects within minutes. **(B)** The surface of the harvested bamboo shoots also attracts some dipetran insects. The most abundant dipteran insects were identified as *Gastrozona fasciventris* **(C)** and *Atherigona orientalis* **(D)**, respectively.

It has been considered that most of the potexviruses are not transmitted by insect vectors (Koenig and Lesemann, 1978), with only few exceptions in earlier archives (Schmutterer, 1961; Goth, 1962; Kassanis and Govier, 1971), including a distinct potexvirus, *Strawberry mild yellow edge associated virus* (SMYEaV) that has been reported to be transmitted by aphids in a persistent mode (Jelkmann et al., 1990), although the involvement of a luteovirus (Yoshikawa et al., 1984; Martin and Converse, 1985; Spiegel et al., 1986) as the helper could not be ruled out (Jelkmann et al., 1990). Similar to most potexviruses, insect-mediated transmission of BaMV has not been reported previously. Thus, the control and prevention of insect pests are not included in the current recommendations for BaMV disease management (e.g., Nelson and Borth, 2011). However, field observations suggested that insect vectors may be involved in BaMV transmission among bamboos. Firstly, the BaMV-infected bamboo clumps in bamboo plantations are not usually distributed closely together. Rather, the patterns of the disease incidence are often sporadic, discontinuous, and not correlated to the route of harvesting or pruning. If BaMV is only transmitted by contaminated tools, one would expect to see disease incidences connected to the sources of contamination or along the paths of maintenance work. Secondly, the cut surfaces of the rootstocks of harvested bamboo shoots (**Figure 1A**) and the pruned culms attract large amounts of insects, mostly of the order *Diptera*, within minutes. The dipterans stay probing and feeding on the cut surfaces till the surfaces dry out if not disturbed, and fly on to the newly generated wounds of the bamboo clumps, possibly far away from the original clump as the harvesting or pruning processes proceed while the insects are feeding. If the dipterans are actually involved in BaMV transmission among bamboos, the lack of measures against insect pests may constitute a loophole in the current integrated disease management of BaMV.

In this study, we aimed to explore the possibility that insects may mediate the transmission of BaMV, a potexvirus, among bamboos. Several lines of evidence were provided in support of the notion that at least two dipteran insects may serve as vectors for transmission of BaMV among bamboos. The results revealed the potential threat of the dipteran insects with sponge-like mouthparts as vectors for at least one plant virus, and suggested that the control of these insects should be integrated into the current systems for disease management against BaMV infections.

## Materials and Methods

### Extraction of Total Nucleic Acids from Bamboo Tissues and Insects

To prevent the contamination of non-specific nucleic acids on the outer surfaces of the insects, RNase Away (Sigma–Aldrich, Shanghai, China) treatment was included as the first step of sample preparation. To determine the efficiency of RNase Away in removing the contaminating nucleic acids, BaMV-free *Gastrozona fasciventris* were anesthetized with acetone vapor and placed into 1.5-ml Eppendorf tubes containing 20 μl of BaMV (0.1 mg/ml in 0.5 M sodium borate buffer, pH 8.0) individually. The tubes were then incubated on ice for 5 min to coat BaMV onto the surfaces of the insects and the tubes. Following the removal of BaMV solution by manual pipetting, RNase Away (200 μl) was added to each tube. The tubes were then incubated at room temperature with gentle shaking for 1, 2, or 3 min, followed by thorough washing with 1 ml of de-ionized H_2_O for three times (3 min each). Then the samples were subjected to RNA extraction and analyses as described below. It was thus determined that the bamboo tissue and insect samples should be treated with RNase Away for 3 min to remove the contaminating nucleic acids on the surfaces before extraction of total nucleic acids (see Results). Total nucleic acids were extracted from bamboo or insect tissues according to the methods described previously (Haible et al., 2006), with minor modifications. Briefly, the RNase Away-treated bamboo (0.1 g leaf or shoot tissue) or insect (whole body) samples were ground to powders in liquid nitrogen and the total nucleic acids were extracted by the addition of 0.2 ml of extraction buffer (100 mM Tris–HCl pH 7.5; 1 mM EDTA; 100 mM NaCl; 100 mM DTT, 0.6% SDS) and equal volume of saturated phenol. After centrifugation at 12000 × *g* for 10 min, the supernatants were transferred to new tubes and the nucleic acids precipitated by the addition of 2.5 volume of 95% ethanol. The final nucleic acid pellets were re-suspended in 20 μl of de-ionized H_2_O for further analyses.

### Molecular Identification of Dipteran Insects and Detection of BaMV RNA by Reverse-Transcription-Polymerase Chain Reaction (RT-PCR)

To identify the dipteran insects collected from bamboo plantations, the chromosomal *cytochrome oxidase I* (*COI*), *28S rDNA* and, mitochondrial *16S rDNA* gene segments were amplified by PCR using gene-specific primer pairs (**Table 1**), cloned, and sequenced following standard protocols described in the Barcode of Life Database (Ratnasingham and Hebert, 2013; Smit et al., 2013). For the detection of BaMV genomic RNA, the aforementioned total nucleic acids (2.5 μl) were used as the template in a mixture (10 μl) containing 50 pmole of oligo dT primer. The template-primer mixtures were heated to 65°C for 2 min, snap-chilled on ice for 1 min, then transferred to a RT reaction containing 10 mM DTT, 0.25 mM dNTPs, and 5 units of SuperScript III reverse transcriptase (Thermo Fisher Scientific, Waltham, MA, USA). The RT reaction mixture was incubated at 37°C for 60 min, followed by inactivation at 85°C for 5 min. The cDNA products were used as the template in the polymerase chain reaction (PCR) for the detection of BaMV in bamboo tissue and dipteran insect samples using BaMV-specific primer pairs (**Table 1**). The PCR mixture (50 μl) contained 5 μl of 10× rTaq buffer, 5 μl of dNTP mixture (2.5 mM each), 0.25 μM of specific primer pair (e.g., B-5981R plus B-6366), 1.0 unit of rTaq DNA polymerase (Toyobo Life Science, Osaka, Japan) and 1–2 μl of the aforementioned total nucleic acids or cDNA products as the template. PCR was performed as follows: reaction mixtures were heated to 94°C for 4 min for initial denaturation, followed by 35 cycles of denaturing at 94°C for 40 s, annealing at 47°C for 45 s, and extension at 72°C for 45 s, with a final extension at 72°C for 20 min. The PCR products were then analyzed by electrophoresis through a 1% agarose gel, stained with ethidium bromide (EtBr), and examined by UV illumination.

**Table 1:** Primers used in this study.

Name	Sequence (5′ to 3′)	Target gene	Reference
FMDV2aF	GGGATCCGCGGCCGCGCT	FMDV 2A peptide	Ryan et al., 1991
	GTTGAATTTTGACTTCTTA		
	AGCTTGCGGG		
FMDV2aR	CCTGGGCCCGGGTCCGGG	FMDV 2A peptide	Ryan et al., 1991
	GTTGGACTCGACGTCTCC		
	CGCAAGCTTAAGAAGG		
GFP F	AAGGATCCATGGTGAGCAAGGG	nts 1-14 of GFP	This study
GFP R	TGGGATCCTTACTTGTACAGCTCG	nts 702-717 of GFP	This study
1R	GAAAACCACTCCAAACG	nts 1-17 of BaMV	This study
331	GGAGATATGAGGCCGTCCG	nts 313-331 of BaMV	This study
2934R	ACTGCATCCAAACCGAAAAC	nts 2934-2953 of BaMV	This study
3527	TCTTGAGACTGGTCATACG	nts 3509-3527 of BaMV	This study
5981R	CACAATATAAATGGTGTGCG	nts 5981-6000 of BaMV	This study
6366	TGGAAAAAACTGTAGAAACCAAAAGG	nts 6341-6366 of BaMV	This study
16S-F	TAGTTTTTTTAGAAATAAATTTAATTTA	16S rDNA	Smith et al., 2003
16S-R	GCCTTCAATTAAAAGACTAA	16S rDNA	Smith et al., 2003
28SS	GACCCGTCTTGAAMCAMGGA	28S rDNA	Asokan et al., 2013
28SA	TCGGARGGAACCAGCTACTA	28S rDNA	Asokan et al., 2013
TEPCOIF	TAAACTTCAGCCATTTAATC	COI	Smit et al., 2013
TEPCOIR	TTTTCCTGATTCTTGTCTAA	COI	Smit et al., 2013

### Purification of Green Fluorescent Protein (GFP)-Tagged BaMV Virions

To track the ingestion of BaMV virions, a mutant BaMV construct that generates GFP-tagged BaMV virions in plants, designated pCB-GFP2a-CP, was constructed based on a mutant infectious clone of BaMV, pBS-d35CP (Yang et al., 2007). The coding sequences of FMDV 2A cotranslational dissociation peptide (LLNFDLLKLAGDVESNPGP) (Ryan et al., 1991) was amplified by PCR with primers FMDV2aF and FMDV2aR (**Table 1**), and inserted to the 5′-end of truncated CP ORF on pBS-d35CP following restriction digestion with *Bam*HI and *Psp*OMI. The GFP coding region (Sheen et al., 1995) was amplified with primers GFP F and GFP R (**Table 1**), digested with *Bam*HI and then inserted to the 5′-terminus of the FMDV 2A coding region to give pCB-GFP2a-CP. Due to the presence of FMDV 2A cotranslational dissociation peptide, the inoculation of pCB-GFP2a-CP in plants would generate both GFP-fused and unfused BaMV CP to assemble into GFP-tagged BaMV virions, together with free GFP-2a fusion proteins. The incorporation of FMDV 2A peptide would increase the assembly efficiency and stability of the GFP-tagged BaMV virions, compared to the construct without FMDV 2A peptide (Hsu et al., unpublished). Leaves of *Chenopodium quinoa* mechanically inoculated with purified plasmid pCB-GFP2a-CP DNA (1 μg/μl in de-ionized water, 10 μl/leaf, 5 leaves/plant) or wild type BaMV virions (0.1 μg/μl in de-ionized water, 10 μl/leaf, 5 leaves/plant) were harvested at 10 dpi. The leaves were ground in de-ionized water (1:10 w/v) to prepare the inoculum used for mass-inoculation of *C. quinoa* plants (50–100 plants/batch). At 10 dpi, the leaves were harvested, and the GFP-tagged or wild type BaMV virions were subsequently purified from the leaves as described by Lin and Chen (1991). The yield was determined spectrophotometrically by absorbance at 280 nm (Lin and Chen, 1991). Purified BaMV virions were dissolved in BE buffer (50 mM Borate, pH 8.0, 1 mM EDTA), then stored at -20°C until used. It should be noted that the purification procedure include an ultracentrifugation through a 5-ml 20% sucrose cushion, which precludes most of the unassembled viral proteins in the pellets (Muthamilselvan et al., 2016). However, the possibility of the presence of some unassembled BaMV CP, GFP-tagged BaMV CP, and GFP-2a fusion proteins in the virion preparations could not be ruled out.

### Dipteran Insect-Mediated Transmission

BaMV-free green bamboo (*Bambusa oldhamii*) seedlings originating from meristem tip-cultured bamboos (Hsu et al., 2000) were kindly provided by Dr. Choun-Sea Lin (Agricultural Biotechnology Research Center, Academia Sinica, Taiwan). Alternatively, BaMV-free green bamboo seedlings propagated by air-layering on BaMV-free bamboo plants were obtained from a bamboo seedling plantation of Mr. Kuo-Chen Chang, which is regularly indexed for BaMV infection. The air-layered bamboo seedlings were used as the hosts for BaMV-transmission assays due to their ability to produce more bamboo shoots. To facilitate the feeding of the flies, the newly emerged bamboo shoots were cross-sectioned at the crown region to mimic the harvesting process. The dipterans were co-incubated with restrained the bamboo seedlings in 200-mesh insect domes for infection assays. For inoculation, 4-7 dipterans fed with liquid medium (10% sucrose, 2% yeast extract) supplemented with purified BaMV virions (0.1 mg/ml, mimicking the concentration of BaMV in bamboos) were released into the insect domes containing bamboo seedlings with wounds. After incubation for 24 h, the dipterans were removed manually, and the bamboo seedlings were maintained in the greenhouse or insect domes for at least 60 days until assayed. To confirm the BaMV-free condition of the test plants, the un-inoculated siblings of the tested bamboo seedlings from the same mother-stocks were assayed for the presence of BaMV following the inoculation assays by northern and western blot analyses.

### Northern, and Western Blot Analyses

To verify the infection and replication of BaMV in bamboo plants, northern and western blot analyses were performed as described by Huang et al. (2012) and Hung et al. (2014), respectively. For northern blot hybridization, ^32^P-labeled probes specific for the 3′ untranslated regions of BaMV RNAs (Hsu et al., 2000) were used to detect the presence of genomic and two major subgenomic RNAs, which are only transcribed following the successful replication of BaMV genomic RNAs. For western blot analysis, specific antibody (Hung et al., 2014) against the triple gene block protein 1 (TGBp1) of BaMV, which is translated from subgenomic RNA 1 and not present in the inoculum, was used to demonstrate successful infection and gene expression of BaMV in inoculated bamboos.

## Results

### BaMV Could be Detected in Two Major Dipteran Insect Species Collected from BaMV-Infected Bamboo Plantations

**FIGURE 2:**
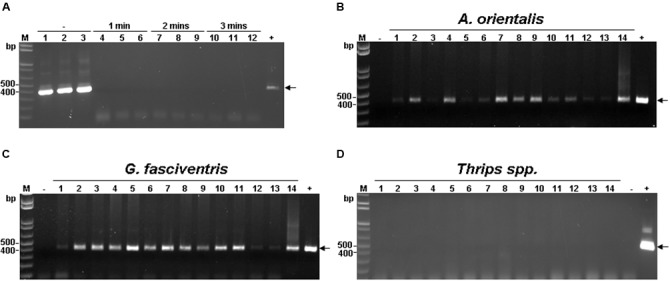
**Detection of BaMV RNA in insects collected from BaMV-infected bamboo plantation. (A)** Determination of RNase-Away treatment time. See Section “Materials and Methods” for details. Briefly, BaMV-coated *G. fasciventris* individuals were treated with RNase-Away for 1–3 min as shown on top of the lanes, or untreated (as indicated by the “–” sign), rinsed thoroughly and tested for presence of BaMV by RT-PCR by primers B-5981R and B-6366 (**Table 1**). The expected position of BaMV-specific products is indicated by the arrow. **(B–D)** Detection of BaMV RNA in insect samples by RT-PCR using the same condition as in **(A)**. Lane M, 1 kb ladder size marker; lanes 1 through 11, *A. orientalis* **(B)**, *G. fasciventris* **(C)** or *Thrips* spp. **(D)** samples; lanes 12, 13, surface and inner tissues of bamboo shoots, respectively; lane 14 bamboo leaf sample collected from BaMV-infected plantation. The plasmid pCB (1 ng, Yang et al., 2007) and total nucelic acids from BaMV-free bamboo samples were used as positive and negative controls (lanes + and –), respectively.

The harvesting, pruning, and other regular maintenance of bamboo crops create cut surfaces or wounds that attract many insects within minutes, mostly in the order *Diptera* (**Figures 1A,B**). Two major insect species were identified to be *Gastrozona fasciventris* (**Figure 1C**) and *Atherigona orientalis* (**Figure 1D**), based on the morphology, wing markings (Permkam, 2005), and nucleotide sequences of the chromosomal *COI*, *28S rDNA* and, mitochondrial *16S rDNA* gene segments (Smith et al., 2003; Ratnasingham and Hebert, 2013; Smit et al., 2013). Other minor species included *Taeniostola vittigera*, *Stypocladius appendiculatus*, and *Drosophila melanogaster*, identified based on the morphologies. To test whether BaMV may have insect vectors, we collected insects from five bamboo plantations (**Table 2**) located in the major bamboo production areas in southern (two plantations in Baihe, Tainan City), central (two plantations in Tanzi, Taichung City), and northern Taiwan (one plantation in Wugu, New Taipei City). The BaMV disease incidences in these plantations ranged from 40 to 70%. RT-PCR was used to detect the presence of BaMV in the main insect species collected. RNase Away was used to avoid the contamination of BaMV on the outer surfaces. To determine the treatment conditions, a pre-test was performed using BaMV-coated *G. fasciventris*. As shown in **Figure 2A**, the treatment of RNase Away for 1 min was enough to remove the contamination of BaMV. For the following experiments, the insect samples were treated with RNase-Away for 3 min to ensure the removal of the possible contaminations of RNases and RNAs, and thoroughly rinsed with RNase-free de-ionized water before being subjected to total nucleic acid extraction. RT was performed using oligo-dT as the sole primer. The cDNA products were then subjected to PCR with primer pair specific to BaMV 3′-terminus (B-5981R and B-6366). The results revealed that BaMV could be detected in the dipteran insect samples, *A. orientalis* and *G. fasciventris*, but not in the thrips (*Scirtothrips dorsalis*) collected from the backside of the leaves of BaMV-infected bamboos (**Figure 2**). The results of the BaMV detection in *A. orientalis* and *G. fasciventris* collected from the five bamboo plantations were summarized in **Table 2**. BaMV detection was not performed on *T. vittigera*, *S. appendiculatus*, and *D. melanogaster*, since these minor insects were not always trapped in all bamboo plantations surveyed, and thus not included in further analyses.

**Table 2:** Detection of BaMV in two major dipterans found in different bamboo plantations in Taiwan.

Location	*Atherigona orientalis*	*Gastrozona fasciventris*
Baihe #1, Tainan City	25/26^a^	18/24
Baihe #2, Tainan City	17/20	5/9
Tanzi #1, Taichung City	1/7	6/14
Tanzi #2, Taichung City	3/8	5/7
Wugu, New Taipei City	10/11	4/4

^a^*Number of BaMV-positive insects/number of tested insects.*

To further explore the possibility that the dipteran insects may mediate the transmission of BaMV, we established BaMV-free colonies of *G. fasciventris* and *A. orientalis* on BaMV-free bamboo seedlings in 200-mesh insect domes. The insects and the bamboo seedlings were indexed bi-weekly to ensure the BaMV-free status (data not shown). These BaMV-free dipteran insects were used in the following assays.

### BaMV Could be Ingested into the Bodies of the Dipteran Insects

**FIGURE 3:**
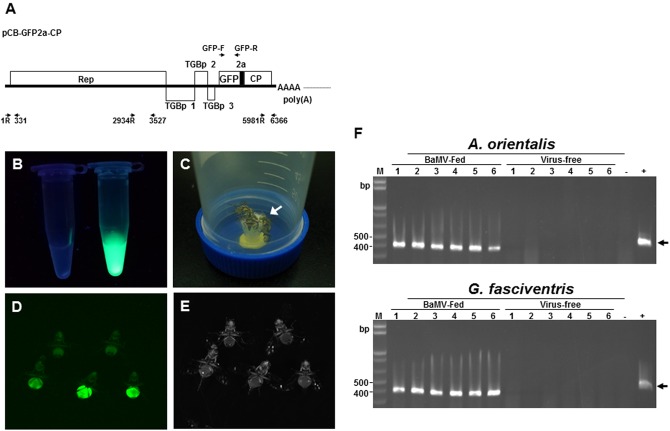
**Tracking the ingestion of BaMV in *G. fasciventris*. (A)** Schematic representation of the plasmid pCB-GFP2a-CP. The coding sequences of FMDV 2A co-translational dissociation peptide (short black box) and GFP inserted at N-terminus of BaMV CP genes. The genome of BaMV is represented by the thick black line. ORFs were shown as boxes with identities indicated. The relative positions and directions of the primers used in **Figure 5** are also shown. **(B)** Purified GFP-tagged BaMV particles (1 mg/ml, tube on the right) under UV illumination (360–390 nm). Equal volume of the wild type BaMV was shown on the left. **(C)** Virus-feeding treatment. Groups of two to seven *G. fasciventris* flies were fed with media containing GFP-tagged BaMV through the cotton matrix (indicated by the arrow) affixed to the cap of the tube. **(D,E)** Examination of the ingestion of GFP-tagged BaMV in *G. fasciventris.* The flies were fed with liquid medium supplemented with wild type BaMV (two flies in the upper row) or GFP-tagged BaMV (three flies in the lower row) for 24 h, anesthetized by acetone vapor, and examined by illumination of blue light (485 nm, **D**) or white light **(E)**. Note that the flies were only slightly anesthetized by acetone for imaging to retain their ability for feeding and flying in the virus-transmission assay, which led to the movement of some of the flies in the matching photos in **(D,E)**. The flies were turned upside down to show the ventral views. **(F)** Detection of BaMV RNA in *A. orientalis* and *G. fasciventris* flies fed with BaMV-containing medium. To test the ability of dipteran insects in acquiring BaMV through feeding, *A. orientalis* and *G fasciventris* flies were fed with BaMV-containing medium (10% sucrose, 2% yeast extract, and 0.01% BaMV) or virus-free medium for 24 h, as indicated on top of the lanes. The presence of BaMV in insect bodies was assayed by RT-PCR as described above.

To explore the relationship between BaMV and the dipterans, GFP-tagged BaMV virions were purified from *C. quinoa* leaves inoculated with pCB-GFP2a-CP (**Figure 3A**), which express GFP-fused BaMV CP. Examination of the purified GFP-tagged BaMV virions under UV illumination confirmed the presence of GFP-tags on the purified virions (**Figure 3B**, tube on the right), as compared to the wild type BaMV virions (**Figure 3B**, tube on the left). The dipterans were fed with liquid medium containing 0.1 mg/ml of purified GFP-tagged or wild type BaMV virions (**Figure 3C**) in a inversed 50-ml Falcon conical tube for 24 h. The presence of GFP signals (likely representing the GFP-tagged BaMV virions) in dipterans were examined by using a LAS-4000 Chemiluminescence and Fluorescence Imaging System (Fujitsu Life Sciences, Tokyo, Japan). The result revealed that green fluorescence was clearly visible in the abdomen portion of *G. fasciventris* fed with GFP-tagged BaMV virions (**Figures 3D,E**, lower row), while those fed with wild type BaMV exhibited only background fluorescence. However, the green fluorescence might also be contributed by the unassembled GFP-tagged BaMV CP or GFP-2a proteins in the virion preparations, thus the presence of BaMV RNAs in the dipterans were further assayed. Following surface decontamination by RNase Away and RNA extraction, the presence of BaMV genomic RNA in *A. orientalis* and *G. fasciventris* fed with BaMV-containing medium was confirmed by RT-PCR (**Figure 3F**). The observations suggested that BaMV may actually enter the digestive systems of dipterans, instead of just temporarily associated with the mouthparts of the insects as in the cases for non-persistent type transmission (Ng and Falk, 2006).

### BaMV May Be Retained Inside the Bodies of *A. orientalis* and *G. fasciventris*

**FIGURE 4:**
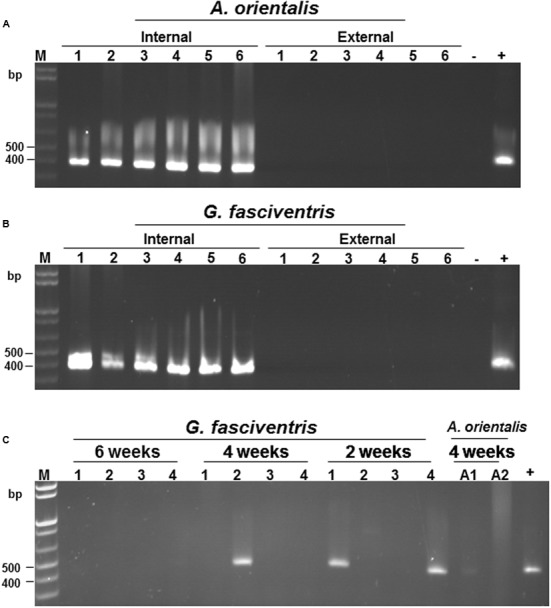
**Verification of the internal localization of BaMV in the dipterans.** Following feeding with BaMV-containing medium for 24 h and virus-free medium for 72 h, the presence of BaMV RNA on the external surfaces (lanes labeled “External”) and within insect bodies (lanes labeled “Internal”) of individual *A. orientalis* **(A)** and *G. fasciventris* **(B)** flies were assayed by RT-PCR as described above. RT-PCR products using nucleic acids extracted from insects fed on healthy bamboo plants or BaMV-containing medium (1 μl) as the templates were used as negative (lanes -) and positive controls (lanes +), respectively. **(C)** Detection of the retention of BaMV within dipteran insects by RT-PCR. The presence of BaMV RNA within *G. fasciventris* samples fed with GFP-tagged BaMV was examined at two-week intervals (indicated on top of the lanes) by RT-PCR as described above (four insects per group). Lanes A1 and A2, *A. orientalis* samples collected at 4 weeks post BaMV-feeding; lane M, size marker, lane +, positive control.

To verify the localization of BaMV on the dipterans, *A. orientalis* and *G. fasciventris* insects were fed with BaMV-containing medium for 24 h as described above, followed by virus-free medium for 72 h. The flies were collected individually in 1.5-ml Eppendorf tubes, and then soaked in nucleic acid extraction buffer (120 μl) with gentle shaking (60 rpm) for 3 min to elute the virus particles possibly adhered to the external surfaces. The eluate was then collected and subjected to total nucleic acid extraction as describe above. The washed insect bodies were centrifuged twice at 1000 × *g* for 10 s to remove excess wash buffer, and also subjected to total nucleic acid extraction. Total nucleic acids from insects fed on healthy plants were used as the templates for the negative controls. The presence of BaMV genomic RNAs was assayed by RT-PCR using primer pair B-5981R and B-6366. The result revealed that BaMV RNAs could only be detected in the insect bodies, not in the eluates of the external portions of *A. orientalis* and *G. fasciventris* (**Figures 4A,B**, respectively). The above observations suggested that BaMV virions are not simply adhering to the surfaces of *A. orientalis* and *G. fasciventris* for dispersion by the mechanical contacts between insects and plants. To test the ability of the dipterans to retain BaMV RNAs, the dipterans were fed with BaMV-containing medium for 24 h, followed by feeding with virus-free medium for up to 6 weeks. Total RNAs were extracted from samples collected at 2-week intervals and subjected to RT-PCR analysis for the presence of BaMV RNA. The results showed that BaMV RNAs could be retained by *G. fasciventris* for up to 4 weeks (**Figure 4C**). On the other hand, the BaMV RNA appeared to be degraded after 4 weeks in *A. orientalis*, but barely detectable amount of RT-PCR products was still observed at least in one of the samples (**Figure 4C**, lane A1).

### BaMV May Survive the Digestive Systems of the Dipterans

**FIGURE 5:**
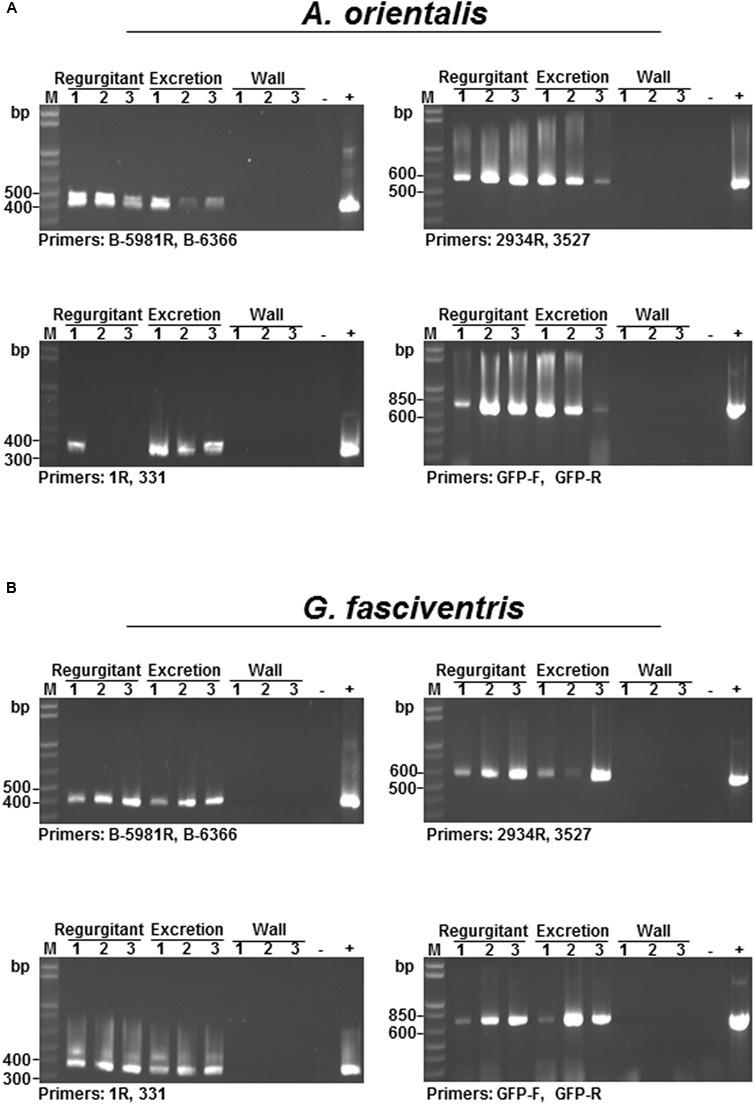
**Detection of GFP-tagged BaMV in the regurgitant and excretion fluids of dipteran insects.** To investigate whether BaMV RNA could pass through the digestive systems of diptera insects, the reguritant and excretion fluids (as indicated on the top of the lanes) were collected from *A. orientalis* **(A)** and *G. fasciventris* **(B)** fed with GFP-tagged BaMV-containing media and subjected to RT-PCR analysis for the presence of BaMV RNA, using different primer pairs (as indicated at the bottom of each gel). To test whether the walls of the feeding tube were contaminated during the feeding process, aliquots of nucleic acid extraction buffer (2 μl) were used to elute materials surrounding the regurgitant or excretion droplets, and subjected to RT-PCR analysis concurrently (lanes labeled “Wall”). For the positive control, 1 μl of GFP-BaMV-supplemented medium was assayed concurrently. Lane M, size marker.

For a virus to be transmitted by an insect vector following ingestion into the abdomen, the virus must survive and pass through the digestive system of the insect. When dipterans feed, they constantly regurgitate crop fluids with digestive enzymes to help dissolve the substrate (Coronado-Gonzalez et al., 2008). The excretions are regularly deposited around the feeding sites. Therefore, the presence of BaMV RNAs in the regurgitants and excretions were examined by RT-PCR to test the survival of BaMV RNA through the digestive system. *A. orientalis* and *G. fasciventris* adults were starved for 4 h, and then fed with media containing GFP-tagged BaMV in 50-ml Falcon conical tubes for 24 h. The regurgitants and excretions of the flies on the wall of the tubes were collected by dissolving in nucleic acid extraction buffer (2 μl) under a dissecting microscope, and subjected to RNA extraction and RT-PCR analysis as described above. Although it has been shown that BaMV RNA could not be detected on the external surfaces of these insects (**Figures 4A,B**), there is still the possibility that the walls of the tubes could be contaminated by GFP-tagged BaMV from the evaporation of the medium or the streaking of the insects. To test the possibility of contamination on the walls, aliquots of 2-μl nucleic acid extraction buffer were used to elute any possible contaminants present on the areas surrounding the regurgitant and excretion droplets. The eluates (lanes labeled “Wall” in **Figure 5**) were then subjected to nucleic acid extraction and RT-PCR analysis concurrently with the regurgitant and excretion samples, serving as negative controls. To overcome the problem of relative inefficient amplification of full-length GFP-tagged BaMV RNAs (∼7.0 kb) directly by RT-PCR using primers 1R and oligo dT, an alternative approach was adopted by using different primer pairs (as shown in **Figure 3A**) to detect different portions of the GFP-tagged BaMV RNA throughout the entire length, using the first-strand cDNA synthesized using the oligo-dT primer as the only template. Since the first strand cDNA is synthesized using oligo-dT primer starting from the 3′-poly(A) tail of BaMV genome, the amplification of the different fragments, especially the one corresponding to the very 5′ terminus (amplified by primers 1R and 331), indicated that the intact BaMV genomic RNA was present in the sample. The results revealed that intact BaMV RNAs could survive the digestive enzymes in the regurgitants and pass through the digestive system to reach the excretion portion of both *A. orientalis* and *G. fasciventris* (**Figure 5**, lanes labeled “Regurgitant” and “Excretion”). In contrast, no BaMV RNA was detected in the eluates from the wall areas surrounding the regurgitant and excretion droplets (**Figure 5**, lanes labeled “Wall”). The above observations raised the possibility that BaMV might be transmitted by the dipterans through the regurgitants or excretions while probing and feeding on bamboos.

### *A. orientalis* and *G. fasciventris* Could Mediate the Transmission of BaMV to Bamboo Seedlings

**FIGURE 6:**
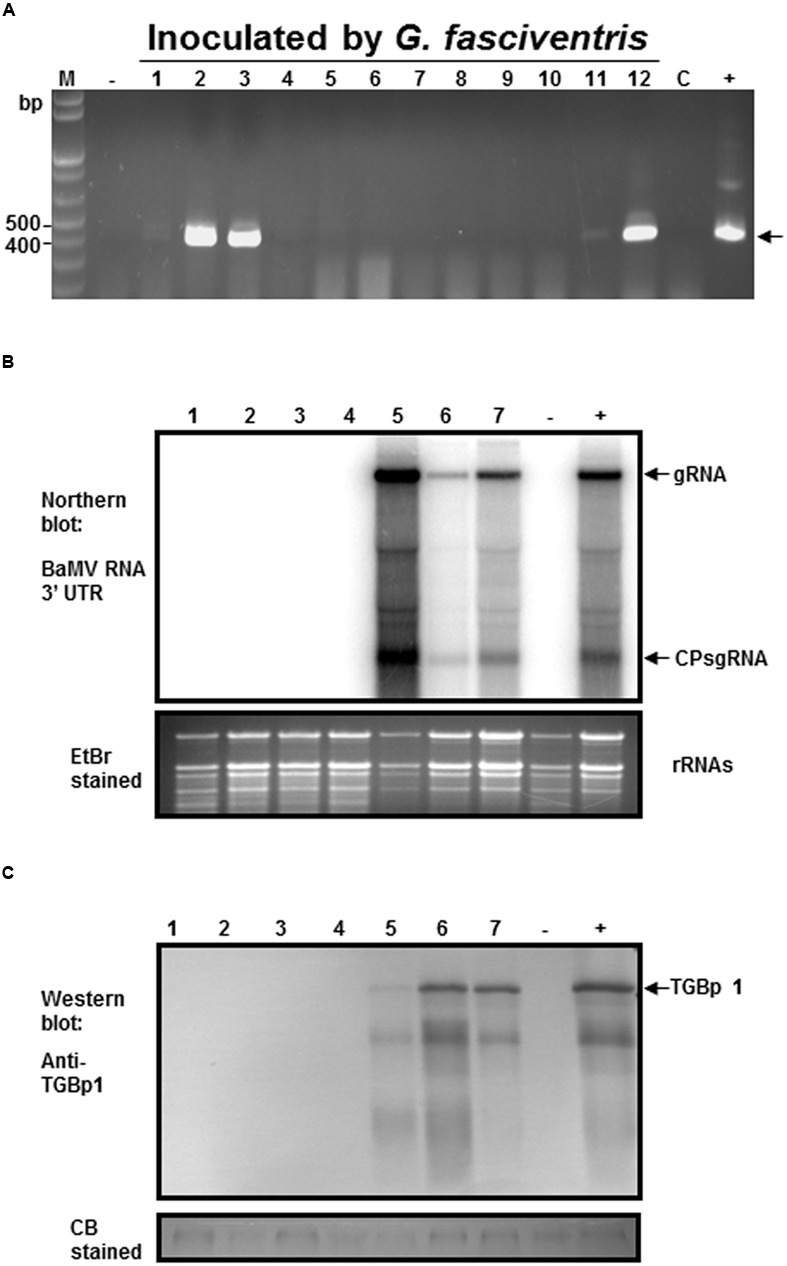
**Analysis of the *G. fasciventris* -mediate BaMV transmission by RT-PCR. (A)** BaMV-free bamboo seedlings were inoculated by *G. fasciventris* fed with BaMV-containing medium in the insect domes. Samples were collected from the newly emerged leaves at the 60th day post inoculation (dpi), and subjected to total nucleic acid extraction and RT-PCR analysis as described above. Lane M, size marker; lane C, sample of cotton balls used for the feeding of the dipterans at the beginning of the experiment; lanes - and +, negative and positive controls as described in **Figure 2**. To verify the inoculation results, northern **(B)** and western blot **(C)** were performed using ^32^P-labeled probes specific for BaMV RNA 3′ untranslated region (Hsu et al., 2000) or antibody against TGBp1 (Hung et al., 2014). Lanes 1–4, RNA or protein extracts of the un-inoculated siblings from the same mother-stock of the bamboo seedlings tested positive in **(A)**; lanes 5–7, RNA or protein extracts from newly emerged leaves of seedlings tested positive for BaMV in panel A (samples #2, #3 and #12, respectively). Lanes - and +, samples from healthy and BaMV-infected bamboo plants, respectively. The positions of BaMV genomic RNA (gRNA), CP subgenomic RNA (CP sgRNA), and TGBp1 are indicated by the arrows.

To test the abilities of the dipterans in transmitting BaMV among bamboo plants, the following experiments were conducted. To simulate the possible insect-mediated transmission during harvesting process, the newly emerged bamboo shoots were cross-sectioned at the crowns to create wounds for insect-mediated virus transmission assays. Since the fusion of the GFP at the N-terminus of BaMV CP interfered with the infectivity in bamboos, the dipterans were fed with liquid media containing purified wild type BaMV virions (0.1 mg/ml) for 24 h, and then subjected to inoculation assays by co-incubation with the bamboo seedlings with cross-sectioned shoots in 200-mesh insect domes. Within each dome, four to seven *A. orientalis* or *G. fasciventris* fed with liquid media containing wild type BaMV were used to inoculate the bamboo seedlings. The dipterans were allowed to feed freely for 24 h, and then removed manually. The newly emerged leaves from the new shoots of the inoculated bamboo seedlings were assayed for the presence of BaMV RNA by RT-PCR at day 60 post inoculation. The results of dipteran insect-mediated BaMV transmission assays were summarized in **Table 3**. The representative result (**Figure 6A** from Experiment 4 in **Table 3**) demonstrated that *G. fasciventris* may actually mediate the transmission of BaMV to bamboo seedlings. To test the possibility of BaMV contamination in the tested plants before the inoculation, we have traced back and examined the un-inoculated siblings from the same mother-stock of the seedlings which tested positive for BaMV after the completion of Experiment 4 (**Table 3**) by northern (**Figure 6B**) and western blot (**Figure 6C**) analyses using ^32^P.-labeled probe specific to the 3′ untranslated region of BaMV RNA (Hsu et al., 2000) and antiserum specific to TGBp1 (Hung et al., 2014), respectively. Three of the inoculated bamboo seedlings (**Figure 6A**, lanes 2, 3, and 12) which tested positive for BaMV in Experiment 4 (**Table 3**) were also assayed concurrently for the products of BaMV infections in the newly emerged leaves. As shown in **Figures 6B,C**, the un-inoculated siblings from the same mother stock (lanes 1–4) remained BaMV-free, while the subgenomic RNAs and TGBp1 protein, which are produced only after the successful infection of BaMV, could be detected in those tested positive (lanes 5–7) in the insect-mediated inoculation assays (**Figure 6A**, lanes 2, 3, and 12).

**Table 3:** Summary of insect transmission assays on bamboos.

Experiment	Bamboo seedling type	Dipteran insect species	# BaMV-infected/# tested plants
1	Air-layering	*G. fasciventris*	1/6
2	Tissue-cultured	*G. fasciventris*	2/12
3	Air-layering	*A. orientalis*	2/14
4	Air-layering	*G. fasciventris*	5/12

## Discussion

### A Novel Finding That Dipterans May Mediate the Transmission of BaMV, a Potexvirus, among Bamboos

Based on the field observations, we explored the possibility of insect-mediated transmission of BaMV among bamboos in this study. Several lines of evidence were provided to support the notion that dipterans may serve as the vector for BaMV, including the association of BaMV with the internal portion of the dipterans, the survival of BaMV RNA through the digestive systems, and the inoculation assays on bamboo seedlings. Our study revealed a novel finding with regards to the role of dipterans as vectors for a plant virus, and the insect-mediated transmission of a potexvirus.

For the role of dipterans as vectors for a plant virus, the flies have been known to transmit many animal pathogens, including viruses (see Baldacchino et al., 2013 for an excellent review), but, to our knowledge, the transmission of a plant virus by dipteran insects with sponge-like mouthparts has not been reported previously. As vectors for animal pathogens, dipterans is believed to transmit viruses by a “mechanical mode,” which appears to occur through either contamination of mouthparts or regurgitation of the contents of the digestive systems onto the openings or wounds of the animals (Baldacchino et al., 2013). This mode of transmission appears to be suitable for plant viruses, especially for those with highly stable virions such as *Tobacco mosaic virus* or *Potato virus X*. However, to our knowledge, no dipterans with sponge-like mouthparts have been reported to transmit plant viruses prior to this study.

As for the insect-mediated transmission of a potexvirus, most potexviruses are not known or thought to be transmitted by insect vectors (Koenig and Lesemann, 1978; Jelkmann et al., 1990). Recent reviews on the insect-transmission of plant viruses did not consider potexviruses in the discussions (e.g., Blanc et al., 2014; Whitfield et al., 2015). In an earlier review (Ng and Falk, 2006), humans are listed as the only rare animal vector for potexvirus transmission. However, *Potato virus X* and *White clover mosaic virus* has been reported to be transmitted by grasshoppers and aphids, respectively (Schmutterer, 1961; Goth, 1962). It has also been reported that *Potato aucuba mosaic virus* could be transmitted by aphids in the presence of a helper virus in the genus *Potyvirus* (Kassanis and Govier, 1971). In addition, the transmission of SMYEaV, by aphids in a persistent mode has been reported (Jelkmann et al., 1990), although it is possible that an SMYE associated luteovirus reported previously (Yoshikawa et al., 1984; Martin and Converse, 1985; Spiegel et al., 1986) may serve as the helper virus for heterologous encapsidation and aphid transmission, or SMYEaV may be aphid-transmitted by other unknown mechanism (Jelkmann et al., 1990). Nevertheless, the transmission of potexviruses by dipterans with sponge-like mouthparts has not been known previously. The findings in this study thus revealed a novel role of the dipterans with sponge-like mouthparts as a vector for a plant virus, at least for a potexvirus, BaMV.

### Relationship between BaMV and Dipterans

The results in this study also provide information for dissecting the relationship in transmission between BaMV and the dipterans. Based on the transmission characteristics, the insect transmission modes of plant viruses have been practically categorized as “non-persistent,” “semi-persistent,” “persistent-circulative,” and “persistent-propagative,” etc. (Ng and Falk, 2006). In this study, we demonstrated that BaMV may actually be ingested into the abdomen (**Figures 3D–F**), remain associated with the dipterans for up to 4 weeks, and survive in the regurgitants and excretions (Figures 4, 5). These results suggested that the relationship between BaMV and these dipterans could be classified as being semi-persistent or persistent (Ng and Falk, 2006). However, it remains unknown whether BaMV may circulate within the hemolymph of the flies and finally into the salivary gland to be regurgitated, or whether BaMV could replicate within the dipterans.

Female phytophagous flies are known to feed by piercing the surface of plants by the ovipositors (as seen on the posterior portion of *G. fasciventris* in **Figure 1C**), then sucking the fluids without laying eggs. During the probing and feeding process, the dipterans with sponge-like mouthparts would regurgitate crop fluid and deposit excretions around the feeding sites (Coronado-Gonzalez et al., 2008). The digestive enzymes in the regurgitants or the excretions may assist in the penetration of cell walls or membranes to facilitate the infection of bamboo tissues by BaMV. This may be one of the possible scenarios how dipterans mediate the transmission of BaMV to bamboos. However, the low infection efficiency in the dipteran insect-mediated transmission assays (14–41%, **Table 3**) and the involvement of artificially created wounds in the assays suggested that the dipteran insects may transmit BaMV in a mechanical-like manner, not directly feeding BaMV into the plants. Further studies are required to fully analyze the relationship between BaMV and the dipterans and the mode of transmission involved.

### The Impact on Current Integrated Pest Management System for BaMV Disease in Bamboos

As mentioned above, no known insect vectors have been reported for BaMV (Elliot and Zettler, 1996; Hsu and Lin, 2004; Nelson and Borth, 2011), thus the current recommendation for the management of BaMV in bamboos did not include the control and prevention of dipterans. The present production systems of BaMV-free bamboo seedlings in Taiwan use indexed bamboo seedlings originated from meristem-tip tissue cultures (Hsu et al., 2000), but the amplification of the original BaMV-free seedlings for downstream growers is dependent on the seedling nurseries in the fields.

The finding in this study that dipterans may mediate the transmission of BaMV highlights the importance of the integration of dipteran insect control and prevention measures into the current disease management system against BaMV. Since the dipterans tested in this study exhibited much longer virus acquisition feeding time, virus retention time, and transmission feeding time, compared to those of the non-persistent mode of transmission, the transmission mode of BaMV by the dipterans may be categorized as at least “semi-persistent,” if not “persistent.” Thus, the dipterans may be controlled by using suitable pesticides, without the concern of causing increased dispersals of the viral diseases as seen for “non-persistent” mode of transmission.

## Conclusion

To our knowledge, this is the first report describing the transmission of a plant virus by dipterans with sponge-like mouthparts, These results expanded the types of insects as vectors for plant viruses, and suggested that dipteran insect control should be concerned and integrated into the disease management measures against viruses, such as BaMV, that are structurally stable enough to survive through the digestive systems of these insects.

## Author Contributions

Study conception and design: Y-HH, N-SL, and C-CH. Acquisition of data: K-CC, L-TC, Y-WH, Y-CL, C-WL, J-TL, and C-CH. Analysis and interpretation of data: Y-HH, N-SL, Y-WH, and C-CH. Drafting and critical revision of the manuscript: Y-HH, N-SL, Y-WH, and C-CH.

## Conflict of Interest Statement

The authors declare that the research was conducted in the absence of any commercial or financial relationships that could be construed as a potential conflict of interest.
